# White matter and cortical gray matter microstructural abnormalities in new daily persistent headache: a NODDI study

**DOI:** 10.1186/s10194-024-01815-1

**Published:** 2024-07-08

**Authors:** Zhilei Li, Yanliang Mei, Wei Wang, Lei Wang, Shouyi Wu, Kaibo Zhang, Dong Qiu, Zhonghua Xiong, Xiaoshuang Li, Ziyu Yuan, Peng Zhang, Mantian Zhang, Qiuling Tong, Zhenchang Zhang, Yonggang Wang

**Affiliations:** 1 Department of Neurology, The Second Hospital of Lanzhou University, Cuiying Gate, No. 82 Linxia Road, Chengguan District, Lanzhou, 730000 China; 2 Headache Center, Department of Neurology, Beijing Tiantan Hospital, Capital Medical University, No.119 South Fourth Ring West Road, Fengtai District, Beijing, 100070 China; 3 Department of Neurology, The First Affiliated Hospital of Wenzhou Medical University, Wenzhou, Zhejiang China

**Keywords:** Newly daily persistent headache, Neurite orientation dispersion and density imaging, Tract-based spatial statistics, Surface-based analysis, Microstructure

## Abstract

**Background:**

New daily persistent headache (NDPH) is a rare primary headache with unclear pathogenesis. Neuroimaging studies of NDPH are limited, and controversy still exists. Diffusion tensor imaging (DTI) is commonly used to study the white matter. However, lacking specificity, the potential pathological mechanisms of white matter microstructural changes remain poorly understood. In addition, the intricacy of gray matter structures impedes the application of the DTI model. Here, we applied an advanced diffusion model of neurite orientation dispersion and density imaging (NODDI) to study the white matter and cortical gray matter microstructure in patients with NDPH.

**Methods:**

This study assessed brain microstructure, including 27 patients with NDPH, and matched 28 healthy controls (HCs) by NODDI. The differences between the two groups were assessed by tract-based spatial statistics (TBSS) and surface-based analysis (SBA), focusing on the NODDI metrics (neurite density index (NDI), orientation dispersion index (ODI), and isotropic volume fraction (ISOVF)). Furthermore, we performed Pearson’s correlation analysis between the NODDI indicators and clinical characteristics.

**Results:**

Compared to HCs, patients with NDPH had a reduction of density and complexity in several fiber tracts. For robust results, the fiber tracts were defined as comprising more than 100 voxels, including bilateral inferior fronto-occipital fasciculus (IFOF), left superior longitudinal fasciculus (SLF) and inferior longitudinal fasciculus (ILF), as well as right corticospinal tract (CST). Moreover, the reduction of neurite density was uncovered in the left superior and middle frontal cortex, left precentral cortex, and right lateral orbitofrontal cortex and insula. There was no correlation between the NODDI metrics of these brain regions and clinical variables or scales of relevance after the Bonferroni correction.

**Conclusions:**

Our research indicated that neurite loss was detected in both white matter and cortical gray matter of patients with NDPH.

## Background

New daily persistent headache (NDPH) is a primary headache distinguished by sudden onset and persistence [1]. Initially described in 1986, it was considered a self-limiting and benign daily headache [2]. However, according to recent findings and the perspective of numerous headache specialists, NDPH is often considered a challenging primary headache to cure [3]. Based on the 3rd edition of the International Classification of Headache Disorders (ICHD-3) criteria, NDPH has distinct features that endure once the headache initiates. They can clearly remember the beginning of headaches and unremitting headaches lasting > 3 months. The pain is akin to migraine-like and tension-type headaches, accompanied by symptoms such as nausea, sensitivity to light, and sensitivity to sound [4]. Previous literature has reported that the etiology of NDPH is possibly related to viral infection [5], immune inflammatory response [6], and excessive movement of cervical joints [7]. Unfortunately, research on the etiology and pathogenesis of NDPH is limited, and our understanding of it remains sparse. Despite its rarity, it is noteworthy that the NDPH is one of the most treatment-resistant primary headaches, resulting in severe disability in patients [1, 3]. There has been a growing interest in NDPH over the past few years, and with the swift advancement of imaging technologies, functional MRI of the brain revealed changes in BOLD signals in the multiple sensory, emotional, affective, and cognitive-related brain regions in patients with NDPH [8–10]. The NDPH has been demonstrated to be related to neuronal activity through functional magnetic resonance imaging. However, the findings on the microstructure of brain tissue in NDPH remain controversial. A literature review found that imaging studies on the brain structure of the NDPH were scant, had a small sample size, and showed significant heterogeneity results [11–14]. This study leverages an advanced higher-order diffusion model that employs more sophisticated algorithms than the traditional DTI model. This approach enables researchers to acquire detailed neural tissue information and precise characterization of neurite morphology characterization while considering biological interpretability [15, 16].

Diffusion tensor imaging (DTI) is an imaging technique that applies a simple data-driven model to indirectly examine the subtle structure of white matter. It normally assesses the integrity of white matter but lacks specificity in neuropathological evaluation. Moreover, DTI is unsuitable for evaluating gray matter due to the intricate orientation of neurites [16, 17]. Newer diffusion magnetic resonance imaging techniques aim to estimate the cellular tissue architecture in vivo, mapping histological features non-invasively, to address these limitations. Neurite orientation dispersion and density imaging (NODDI) is a biophysical model that is arguably the most popular currently [15, 16].

The NODDI has been identified as combining diffusion signals with tissue features via a biophysical mode. This advanced technique quantifies neurite morphology in both white matter (WM) and gray matter (GM), thereby increasing the specificity of diffusion imaging [18, 19]. Within the NODDI model, each voxel is approximated to three main compartments, including an intra-neurite compartment with restricted anisotropic non-Gaussian diffusion (neurite density index (NDI)), an extra-neurite compartment with constrained anisotropic Gaussian diffusion (orientation dispersion index (ODI)), and a water compartment with free Gaussian and isotropic diffusion (isotropic volume fraction (ISOVF)) [15, 20]. NODDI enables the estimation of three critical components of neural tissue at each voxel: NDI, which quantifies the density of axons and dendrites; ODI, which estimates the orientation consistency of neurites; and ISOVF, which calculates the extent of CSF contamination [15, 21]. In general, a lower NDI value indicates loss or damage of neurites. A higher ODI value suggests the crossing of neurites, and a higher ISOVF value can be interpreted as neuroinflammation [16, 19, 21]. The NODDI has three advantages over a conventional model of DTI: i) the assessment of both neurite density and orientation dispersion; ii) the ability to simulate and study the diffusion signal in white matter and gray matter; iii) the minimization of volume effects [18, 22].

Tract-based spatial statistics (TBSS) is a holistic brain voxel analytical approach that synthesizes the benefits of voxel-based analyses and tractography-based analyses. It addresses the inherent alignment and smooth kernel issues found in the voxel-based morphometry, thereby improving sensitivity, reliability, and biological interpretability of the analysis of multisubject diffusion MRI studies [23]. That is why it has been widely applied to analyze changes in white matter. Microstructural plasticity changes in the cortex were assessed vertex by vertex using surface-based analysis (SBA). Compared to traditional voxel-wise volumetric methods, this approach possesses two potential advantages: i) it maintains the spatial continuity of cortical folding; ii) it safeguards against the interference of data originating from non-cortical areas such as WM and cerebrospinal fluid (CSF) [24].

Neurites are essential components that link neurons with synapses, serving as pivotal conduits for transmitting information. Their integrity and function are critical in disease progression, particularly where alterations in damage or plasticity are involved. Many neuropsychiatric disorders, including the migraine and chronic pain, are thought to involve altered neuroplasticity [25, 26]. Using Golgi staining and neurite tracers, neuronal sophistication was reduced in animal models of chronic migraine. Neurite growth was reduced in some regions associated with headache processing. Importantly, a calcitonin gene-related peptide receptor antagonist reversed the attenuated neuronal intricacy induced by nitroglycerin [27]. Effective management of migraines may encompass the medications that promote the restoration of neuronal plasticity. The neurites, as the main channel for information exchange between neurons and synapses, are critical in the pathological mechanism of headaches. Recently, studies have reported using NODDI to detect white matter microstructural changes in migraineurs [28, 29]. These findings indicate that neurite dispersion is damaged in patients with migraine. We investigated pathological features of NDPH for a maiden attempt in white matter and cortical microstructure using the neurite imaging technique. With the novel method of NODDI, abnormal neurite has been demonstrated in many neurological and psychiatric conditions, including multiple sclerosis [30], Alzheimer’s [20, 31], schizophrenia [32, 33], and so on. The robustness of the NODDI model is verified by animal and postmortem experiments [34–37].

Overall, the neurite imaging technique has been applied to comprehensively analyze characteristics of neurites in both white matter and cortex of NDPH and matched HCs. We hypothesized that abnormal neurites existed in WM and GM in the NDPH group. Additionally, we explored whether abnormalities in the microstructure were associated with the clinical features of patients with NDPH, aiming to illustrate pathophysiology and identify the neuroimaging biomarkers and neuromodulation targets in patients with NDPH.

## Method

### Study participants

Between January 2021 and April 2023, 30 individuals diagnosed with NDPH and 32 HCs were enrolled in the Headache Department at the Neurology Centre, Beijing Tiantan Hospital, Capital Medical University. Ultimately, 55 right-handed participants, consisting of patients (*n* = 27) and HCs (*n* = 28), were incorporated into the current study. Inclusion criteria for NDPH included: (1) Both senior neurologists identified patients’ diagnoses according to the ICHD-3 [4]. (2) Participants’ ages ranged from 14 to 60 years. (3) Individuals with NDPH were not experiencing medication overuse or using any preventive headache drugs. (4) None of the patients had NDPH onset after COVID-19 infection. Exclusion criteria encompassed: (1) other types of headaches; (2) associated with neurological or other systemic illnesses; (3) NDPH with explicit secondary factors; (4) with a prior history of drug or alcohol abuse; (5) not compliant with the MRI safety criteria; (6) during pregnancy or breastfeeding; (7) poor and missing data.

Moreover, HCs matched were enrolled. The inclusion criteria for HCs comprised: (1) absence of headache history or family history; (2) matched in age and gender to patients with NDPH. Exclusion criteria comprised: (1) major systemic disorders; (2) during pregnancy or breastfeeding; (3) claustrophobia or metal implants within the body; (4) poor and missing data.

The clinical study was authorized by the research ethics committee of the Headache Center, Department of Neurology, Beijing Tiantan Hospital, Capital Medical University, China, and signed approval was obtained from each participant. The investigation was enrolled on https://www.clinicaltrials.gov (one-of-a-kind identifier: NCT05334927).

We recorded demographic information for all participants. Before conducting the 3.0 Tesla MRI scans, we gathered the clinical data from patients using several assessment scales. These included Visual Analog Scale (VAS) [38], the Patient Health Questionnaire-9 (PHQ-9) [39], the Headache Impact Test-6 (HIT-6) [40], the Generalized Anxiety Disorder-7 (GAD-7) [41], Pittsburgh Sleep Quality Index (PSQI) [42], and Montreal Cognitive Assessment (MoCA) [43].

### Image acquisition

The 3D T1 structural and diffusion MRI images were acquired using the GE 3.0 Tesla MR scanner (Signa Premier, GE Healthcare) with a 48-channel head coil at the National Neurological Center of Beijing Tiantan Hospital. The T1-weighted images were as following settings: MP-RAGE sequence, preparation time = 880 ms, recovery time = 400 ms, acceleration factor = 2, acquisition time = 4:00, field of view = 250 × 250 mm^2^, flip angle = 8°, slices = 192, and 1 × 1 × 1mm^3^ of spatial resolution. DWI was performed with the following parameters: repetition time = 5285 ms, echo time = 85 ms, data matrix = 104 × 104, field of view = 208 × 208 mm^2^, slice thickness = 2 mm, slices = 78, gradient direction = 108, and diffusion sensitivity coefficients (b) = 0, 1000, and 2000s/mm^2^, 50 diffusion-weighted directions at each b-value and 9 b0 scans.

### MRI data processing

The image manipulation includes data preprocessing, brain tissue segmentation, and NODDI fitting. Before preprocessing, both experienced neuroradiologists visually examined each participant’s diffusion and T1-weighted images to ensure they were free from obvious artifacts (e.g., head motion). The diffusion-weighted images were initially processed through the FMRIB Software Library (FSL, version 6.0.1; http://www.fmrib.ox.ac.uk/fsl), which involved rectification of eddy current artifacts, reduction of noises, rectification of inter-volume head motion artifacts, and extraction of brain tissue. Subsequently, the resulting images were calculated through the NODDI MATLAB Toolbox (version 1.0.5; https://www.nitrc.org/projects/noddi_toolbox) with default parameters, which yielded maps of NDI, ODI, and ISOVF. The cortical surface of the T1 structural image was reconstructed using Freesurfer version 6.0. In the image preprocessing stage, tasks such as skull stripping, brain tissue segmentation, cortical reconstruction, and inflation of the cortical surface were conducted. Quality assurance encompassed reviewing each slice individually and performing manual adjustments when necessary.

### Tract-based spatial statistical analysis

The diffusion data underwent voxel-by-voxel statistical analysis using TBSS, which was implemented in FSL. The DTIFIT toolbox within FSL was employed to fit the pre-processed image and generate the FA map based on the DTI model. Using a threshold of 0.2 for FA, the average FA image was computed from all participants’ images. The mean FA skeleton was generated through the thinning FA image. The tbss_non_FA script generated NDI, ODI, and ISOVF skeletons. The white matter skeleton comprised voxels situated solely within the core of the white matter skeleton, excluding the voxels that might contain signals from neighboring tissues. Permutation-based non-parametric analysis was conducted within the white matter skeleton using the FSL randomize command for voxel-wise statistical analyses, with gender and age used as covariates in this study. The threshold-free cluster enhancement (TFCE) and 5000 permutations were utilized to obtain a corrected P-value. Statistical significance for the white matter voxels was determined at a corrected P-value<0.05 following adjustment for multiple comparisons using the family-wise error (FWE) rate control.

### Surface-based analysis

We evaluated the changes in the subtle structure of the cortex by SBA. A 6-degrees-of-freedom (DOF) boundary-based registration (BBR) was employed to register the NODDI maps to T1 structural images. Then, to reduce the risk of contamination, we created and utilized a mid-thickness surface. The NODDI metrics (NDI, ODI, ISOVF) were projected onto the mid-thickness surface. Finally, the resampling of projected maps into template surface space was conducted for further analysis. Before the statistical analysis, all surface maps in this space were smoothed using a Gaussian kernel filter with a full width at half maximum (FWHM) of 10 mm [44–46]. After projecting the relevant metrics information from cortical voxels, the smoothing procedure is performed on the surface, enhancing the statistics’ accuracy.

### Statistical analysis

Statistical analysis was conducted using the IBM SPSS Statistics 25 software package. For the demographic and clinical data, independent sample t-tests were used to analyze continuous variables, while chi-square tests were utilized for categorical variables. Before conducting an independent sample t-test, normality was assessed using the Shapiro-Wilk test. A permutation-based statistical inference tool called “randomize”, implemented in the FSL Randomize tool (version 2.1), was utilized to compare voxel-wise TBSS differences in NDI, ODI, and ISOVF values of white matter between the different groups and threshold-free cluster enhancement (TFCE). The permutation of 5000 iterations ensured robust statistical inference. The threshold for significance in the resulting statistical maps, corrected for multiple comparisons (two-sided, family-wise error (FWE) corrected), was set at *p* < 0.05. Differences in cortical NODDI metrics between NDPH and HCs were analyzed by fitting a generalized linear model (GLM) in Freesurfer using the mri_glmfit tool. For SBA, an uncorrected threshold of *p* < 0.01 was applied to initiate comparison for each vertex. Correction for multiple comparisons was applied to all results by Monte Carlo simulations with 10,000 iterations (using the function mri_glmfit-sim), corrected for two hemispheres, and a cluster-wise threshold of *P* < 0.05. In the GLM, age and gender were incorporated as covariates, and the statistics were adjusted for multiple comparisons. Visualizations of positive results were presented on inflated surfaces of the brain cortex. Correlations between the NODDI metrics and clinical characteristics (duration of NDPH in years, VAS scores, HIT-6 scores, PHQ-9 scores, GAD-7 scores, PSQI scores, and MoCA scores) were assessed through Pearson’s correlation analysis. Significance testing was conducted utilizing the Bonferroni correction method, with a significance threshold of *p* < 0.05/N (where N represents the number of correlation analyses performed).

## Results

### Demographics and clinical characteristics

**Fig. 1:**
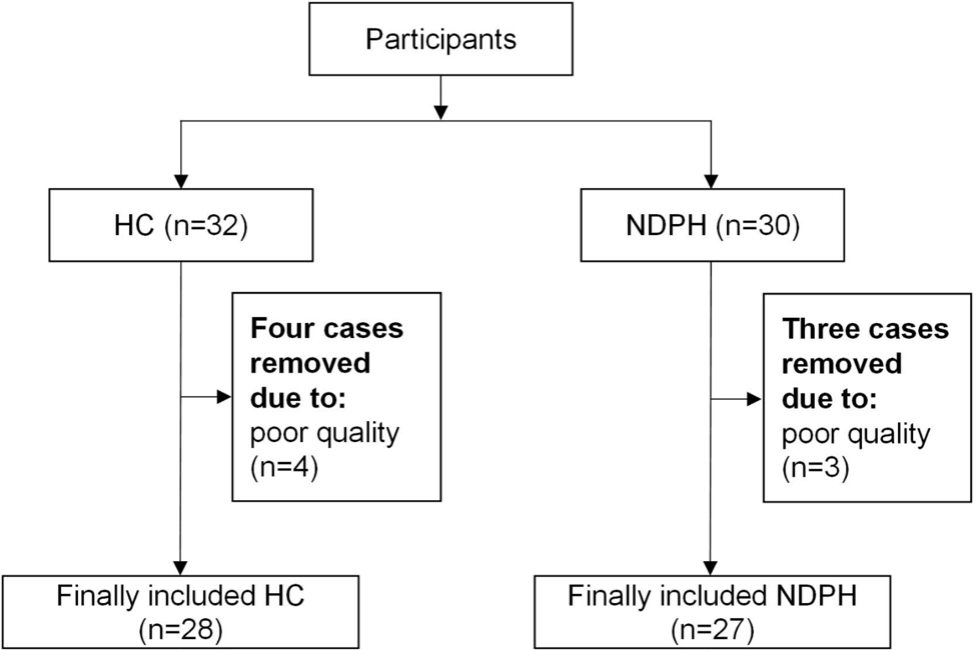
Participant inclusion-exclusion process flowchart. NDPH, new daily persistent headache; HC, healthy control

During the study period, 32 healthy individuals and 30 patients with NDPH were enrolled, meeting the inclusion and exclusion criteria. All data was quality-controlled to rule out participants with poor-quality data, including three patients and four healthy controls. Finally, 27 NDPH and 28 HCs were incorporated in this research (Fig. 1). Descriptive statistics of clinical variables for the patients with NDPH are displayed in Table 1. Age, BMI, and gender ratio did not show significant differences between the two groups.

**Table 1:** Clinical and demographic features of participants

	Controls(*n* = 28)	NDPH(*n* = 27)	*P*-value
Ages (years)	29.54 ± 5.59	28.26 ± 14.64	0.127
BMI	22.34 ± 3.67	23.55 ± 4.29	0.220
Gender (female/male)	17/11	13/14	0.349
Headache history (years)	NA	9.32 ± 10.53	NA
Pain intensity VAS score	NA	5.59 ± 2.15	NA
HIT-6 score	NA	62.63 ± 10.51	NA
PHQ-9 score	NA	10.63 ± 6.43	NA
GAD-7 score	NA	8.44 ± 4.06	NA
PSQI score	NA	9.58 ± 5.35	NA
MoCA score	NA	26.52 ± 3.65	NA

*Note* NDPH, New Daily Persistent Headache; HC, Healthy Control; NA, Not Applicable; BMI, Body Mass Index; VAS, Visual Analogue Scale; PHQ-9, Patient Health Questionnaire-9; GAD-7, Generalized Anxiety Disorder-7; PSQI, Pittsburgh Sleep Quality Index; HIT-6, Headache Impact Test-6; Montreal Cognitive Assessment (MoCA)

### TBSS analysis of NODDI metrics

**Fig. 2:**
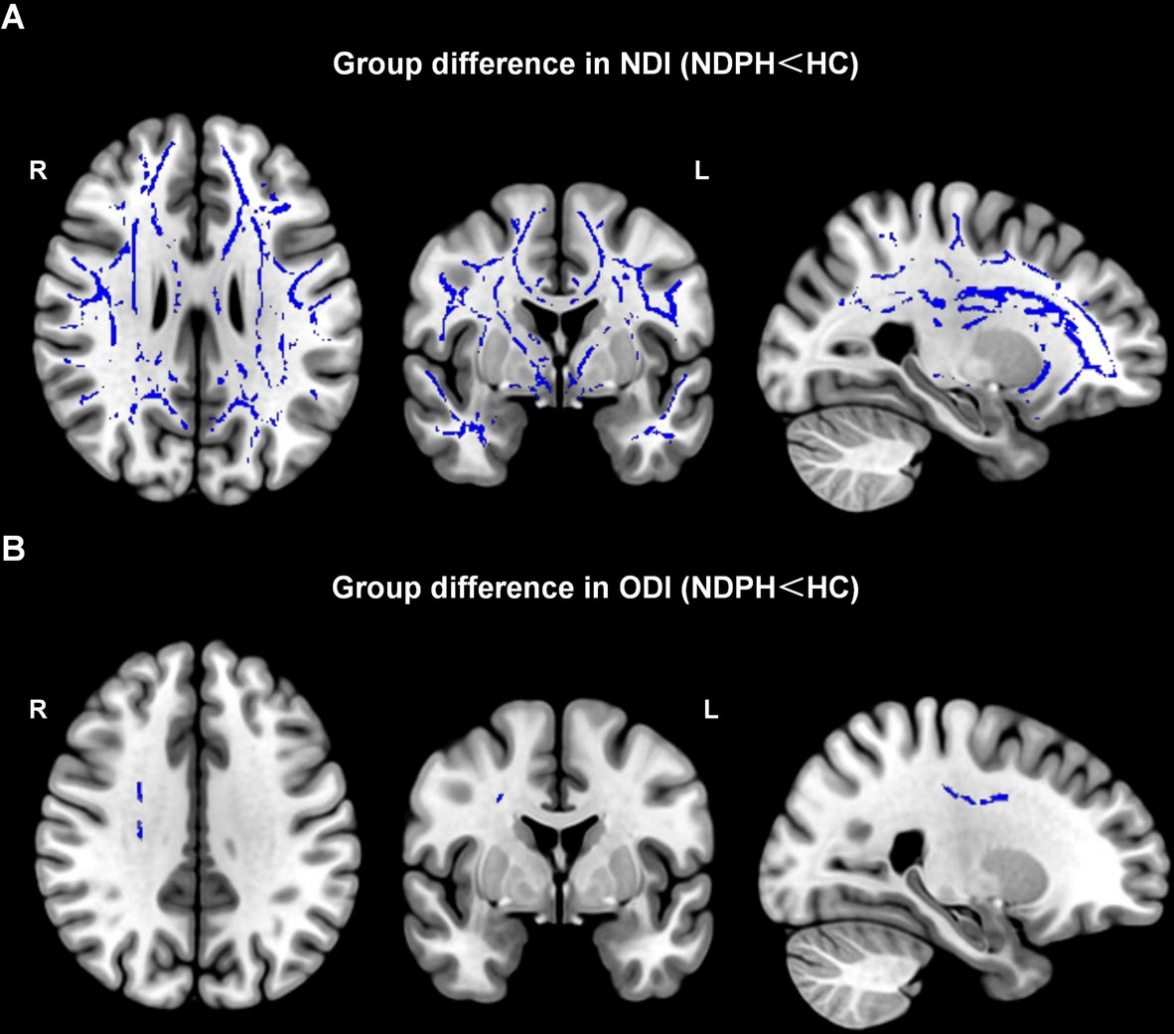
(**A**) White matter regions (blue) showed decreased NDI in patients with NDPH compared to the HCs group (P_FWE_ < 0.05). (**B**) White matter regions (blue) showed decreased ODI in patients with NDPH compared to the HCs group (P_FWE_ < 0.05). NDPH, new daily persistent headache; HC, healthy control; NDI, neurite density index; ODI, orientation dispersion index; L, left; R, right

The TBSS was employed for voxel-wise statistical analysis of NODDI metrics. Compared to HCs, patients with NDPH had significantly lower NDI and ODI in multiple white matter fibers (FWE < 0.05). There was no difference in ISOVF between the two groups. Drawing on insights from prior research, we utilized JHU DTI-based white-matter atlases to present our findings. White matter with reduced neurite density includes bilateral corpus callosum (CC), bilateral corticospinal tract (CST), bilateral anterior thalamic radiation (ATR), right cerebral peduncle (CP), bilateral anterior limb of internal capsule (IC), bilateral retrolenticular part of internal capsule (IC), bilateral posterior limb of internal capsule (IC), bilateral corona radiata (CR), bilateral sagittal stratum (SS), bilateral external capsule (EC), bilateral cingulum, bilateral forceps major (FM), bilateral forceps minor (FM), bilateral fornix/stria terminalis, bilateral superior longitudinal fasciculus (SLF), bilateral superior fronto-occipital fasciculus (SFOF), bilateral inferior longitudinal fasciculus (ILF), bilateral inferior fronto-occipital fasciculus (IFOF) and bilateral uncinate fasciculus (UF) (Fig. 2A). These white matter tracts with reduced ODI include the right corticospinal tract (CST), right anterior thalamic radiation (ATR), right superior corona radiata (SCR), and right superior longitudinal fasciculus (SLF) (Fig. 2B). We focus on these white matter fiber tracts, where the clusters consist of more than 100 voxels (Table 2).

**Table 2:** White matter regions of statistically significant differences between NDPH and HCs

Metric	Contrast	Cluster	Region	Side	Voxels	*P*-value	MNI coordinate			Category
							x	y	z	
NDI	NDPH< HC									
		Cluster1	IFOF	R	57,998	*p*<0.001	38	24	-10	Long associative fiber
		Cluster2			983	0.039	-41	-22	-18	Long associative fiber
			ILF	L						
			IFOF	L						
		Cluster4			147	0.023	-52	-50	-9	Long associative fiber
			SLF	L						
			ILF	L						Long associative fiber
ODI	NDPH< HC									
		Cluster1	CST	R	212	0.041	22	-29	42	Projection fiber

*Note* Inferior Frontooccipital Fasciculus (IFOF), Inferior Longitudinal Fasciculus (ILF), Superior Longitudinal Fasciculus (SLF), Corticospinal Tract (CST); NDPH, New Daily Persistent Headache; HC, Healthy Control; NDI, Neurite Density Index; ODI, Orientation Dispersion Index. Regions with clusters greater than 100 voxels were contained in this table L left and R right

### Surface-based analysis of NODDI metrics

**Fig. 3:**
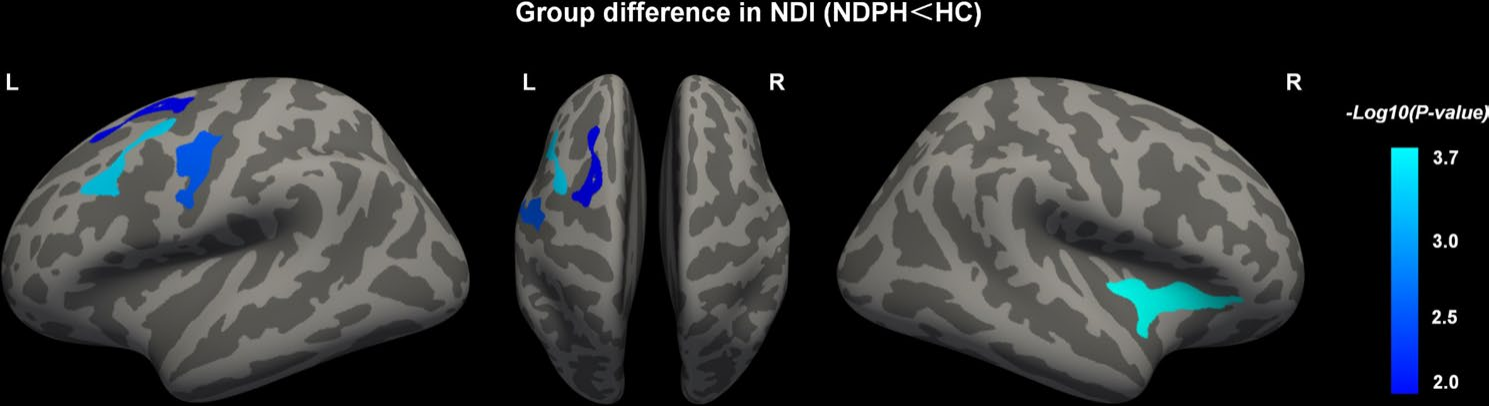
Gray matter regions (blue) showed decreased NDI in the NDPH group compared to the HCs group. NDPH, new daily persistent headache; HC, healthy control; NDI, neurite density index; L, left; R, right

**Fig. 4:**
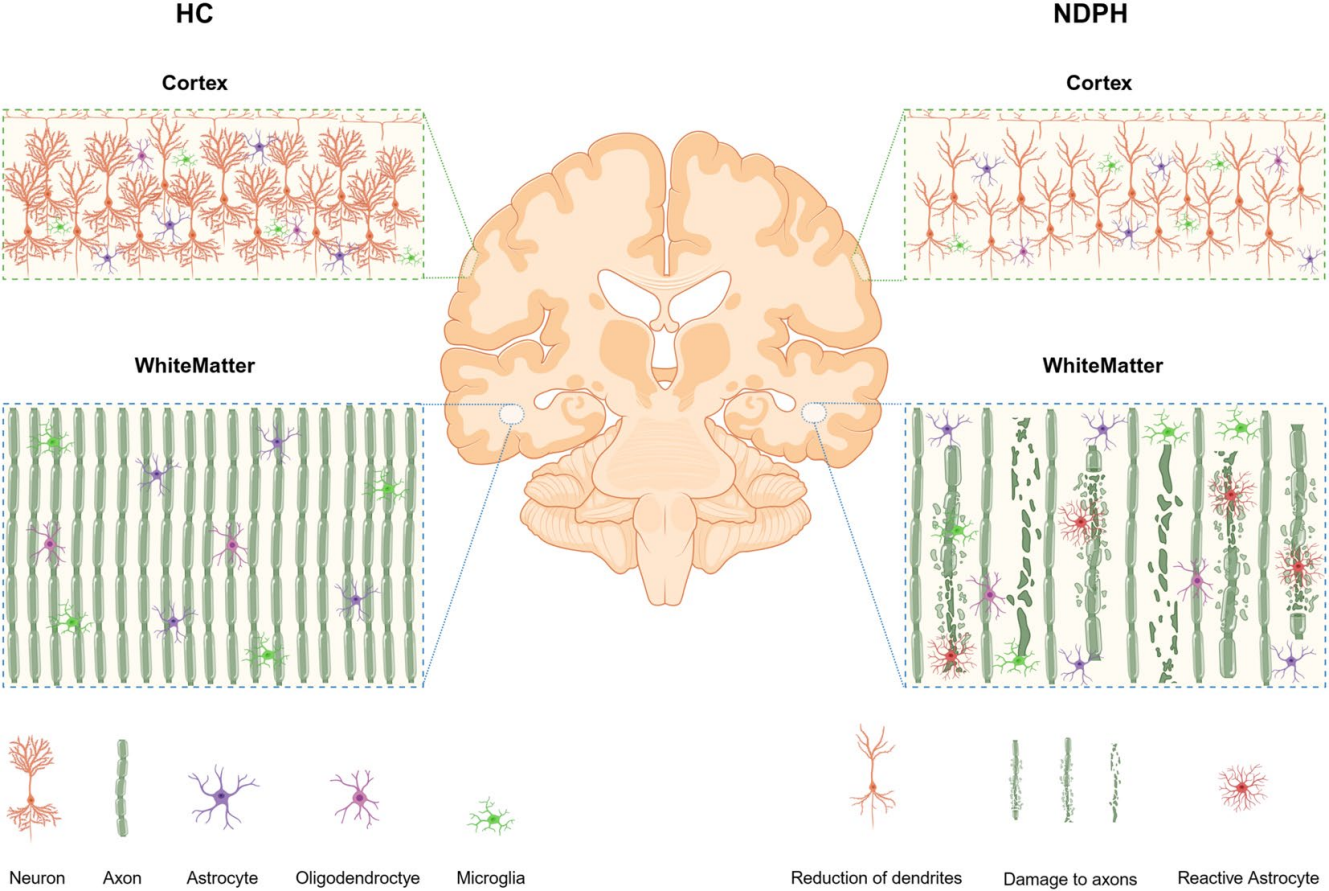
This is an overview of the neurite’s pathological features in the different tissues between NDPH and HCs by NODDI. Axonal damage is prominent in white matter, and a dendrite is lost in the cortex. It was created with http://www.biorender.com/. HC, Health control; NDPH, New daily persistent headache; WM, White matter; GM, Gray matter

We found notable discrepancies regarding NDI between the two groups using a surface analysis method. Compared to the health controls, patients diagnosed with NDPH displayed diminished NDI in the left superior frontal cortex, left middle frontal cortex, left precentral cortex, and right lateral orbitofrontal cortex and insular (Fig. 3; Table 3). There were no significant differences between the two groups regarding ODI and ISOVF (Fig. 4).

**Table 3:** Brain region with NDI changes in NDPH

Brain region	Side	MNI coordinates			Cluster sizes (mm^2^)	CWP
		x	y	z		
Middle frontal cortex	L	-35.6	1.8	51.4	588.81	0.001**
Precentral cortex	L	-54.5	-5.5	39.6	569.30	0.002**
Superior frontal cortex	L	-19.9	5.7	56.7	483.36	0.007**
Lateral orbitofrontal cortex and insula	R	29.8	23.5	3.1	749.46	<0.001**

*Note* The Desikan-Killiany atlas was used to localize brain regions. MNI, Montreal Neurological Institute; NDPH, New Daily Persistent Headache; NDI, Neurite Density Index; L, Left; R, Right. CWP, Cluster-Wise Corrected p-value **, *p* < 0.01

### Correlation analysis

To conduct Pearson’s correlation analysis with clinical variables, we extracted NDI and ODI values from the white matter region (Table 2) and NDI values from the cortical gray matter areas (Table 3). Following adjustment for the multiple comparisons using Bonferroni correction, no statistically significant correlations (*P* > 0.05) were observed between the regional NDI and ODI values and clinical characteristics.

## Discussion

Neurite imaging was applied to examine the brain microstructure of patients diagnosed with NDPH, focusing on white matter and cortical gray matter. However, the ultimate goal of the study is to explore potential pathological mechanisms, identify neuroimaging biomarkers, and pinpoint targets for neuromodulation. The findings revealed a significant decrease in NDI and ODI levels within the white matter of patients diagnosed with NDPH. Furthermore, it was noteworthy that the NDI levels were significantly diminished in the left superior and middle frontal cortex, left precentral cortex, right lateral orbitofrontal cortex, and insula in the NDPH group. Unfortunately, we did not find correlations between clinical features and the positive result. In short, neurite loss is a pathological feature of NDPH compared to HCs (Fig. 4).

### Previous brain structural studies of NDPH

Given the extremely low incidence of NDPH, there were a few imaging studies in the forepassed literature. In a retrospective brain magnetic resonance imaging (MRI) investigation of 97 NDPH patients, only thirteen showed white matter abnormalities and no infarct-like lesions were detected [13]. Another MRI study based on voxel-wise and surface morphometry showed no gray matter abnormalities between NDPH and HCs [12]. However, brain regions’ structure and function alterations were documented in adolescents with NDPH [11]. A recent DTI investigation utilizing TBSS analysis suggested that individuals with NDPH had extensive disruptions in white matter, indirectly implying axonal injury. However, the detailed information regarding the specific tissue was still unknown [14]. The microstructure of white matter in NDPH remains unclear. Additionally, more studies need to be conducted to pay close attention to the cortical microstructure of NDPH. We applied an advanced imaging technique that could characterize neurites to probe into the microstructural changes of white matter and cortex in patients with NDPH.

### Abnormal neurites in the white matter of NDPH

In our research, TBSS analysis found that NDPH had an axonal reduction in several fiber tracts. Meanwhile, we found that damaged fiber bundles were mainly long-range association fibers and projection fibers, which comprised bilateral IFOF, left ILF, left SLF, and right CST. The NODDI offered a more unequivocal interpretation of the changes in the white matter microstructure associated with NDPH compared to conventional methods by distinguishing between the neurite density and orientation dispersion.

The inferior fronto-occipital fasciculus (IFOF) is a major long-range association fiber in the brain. It connects the frontal, temporo-basal, and superior parietal regions with the occipital cortex. The IFOF plays a role in various cognition, including reading, attention, and visual processing [47]. Due to its extensive length, it was vulnerable to damage. The reduction of NDI within the IFOF indicated disrupted information transfer from the occipital cortex to other brain areas, likely attributed to axonal loss or damage. Such disruption could induce elevated excitability within the occipital cortex. It has been believed that the excitability of the occipital cortex easily evokes cortical spreading depression (CSD), leading to headache onset. With the occurrence of CSD, there will be a progressive spread of depolarization attributed to continuous suppression of neural activity, which has been deemed to be a potential mechanism for migraine [48]. Recently, Mendelian randomization analysis suggested that an increased mode of anisotropy (MO) in the left IFOF increases the risk of migraine [29]. It indicates that the IFOF plays a crucial role in migraine pain processing. In our study, the IFOF was the fiber tract with the most pronounced differences between the groups. Therefore, we speculate that impairment of the IFOF may increase susceptibility to CSD, which may lead to continuous headaches of NDPH. Besides, the IFOF is involved in many cognitive functions, which may explain the manifestation of hypomnesia, inattention, and cognitive dysfunction in patients with NDPH.

The superior longitudinal fasciculus (SLF) interconnects the frontal and parietal regions [49]. Its main function includes language processing and visuospatial attention [50]. There is the part of this fiber that connects Broca’s area with Wernicke’s. It suggested that the SLF that was especially left was crucial for language processing [51, 52]. As observed in migraine [53], individuals diagnosed with NDPH were distinct from significant aphasia; however, they had a decline in verbal memory and skills compared to healthy individuals. The research on the vestibular network has shown that the SLF controls vestibular function [54]. Impaired SLF may be responsible for vestibular symptoms observed in individuals with NDPH. They are more likely to suffer vertigo and dizziness than the general population. The research has shown alterations of SLF in migraines combined with depression and anxiety. Abnormalities of these fiber bundles have also been observed in patients with depression and anxiety [55, 56]. Reduction in axonal density in the SLF may be related to anxiety and depression states due to persistent headaches.

The inferior longitudinal fasciculus (ILF) contains these fibers that establish the connections between occipital and temporal regions while traveling across the amygdala, hippocampus, and parahippocampal regions [57, 58]. The ILF potentially affected various brain functions associated with visual processing, encompassing facial recognition, spatial perception, language comprehension, reading ability, emotional response, and visual memory [57, 59]. Meanwhile, the ILF connects important regions for interpreting and perceiving pain. In a DTI study evaluating the integrity of white matter bundles in migraine, disturbances in the ILF were observed [60]. Another structure imaging study of chronic pain has shown that the increased FA is detected in the bilateral inferior longitudinal fasciculi and correlates with pain severity [61]. Some patients with NDPH suffer from panic attacks, which might be linked to the disruption of the connections between the ILF and the amygdala. The ILF has fewer axons in the NDPH group, which may be related to the processing of headaches and complicated perception.

The corticospinal tracts (CST) are probably the best-known white matter tracts. They are projection rather than association fibers. The CST arises from the primary and secondary motor and somatosensory cortices and protracts into the spinal cord. It serves as a descending fiber crucial for nociceptive perception [60]. It has been demonstrated that individuals experiencing chronic pain exhibit elevated axial diffusivity (AD) in certain regions of the CST, indicating compromised tract integrity [62]. The resting-state functional MRI results of migraineurs indicated that the changed functional connectivity was discovered in the periaqueductal gray and precentral region. Furthermore, it demonstrated increased responsiveness in the primary motor cortex and corticospinal tract of migraineurs when using transcranial magnetic stimulation [63, 64]. The current study indicated decreased ODI in the right CST in the NDPH group. That may be due to persistent headaches, which result in reduced fiber complexity.

### Abnormal neurites in the cortical gray matter of NDPH

Cerebral cortical microstructure is fundamental to comprehending pain mechanisms. It is critical to integrate diverse sensory inputs, including processing and regulating pain information transmission [65]. In this study, we pioneered the application of advanced neurite imaging to investigate the morphological pathological features of NDPH in the cortical microstructures. Surprisingly, we observed significant neurite loss in several brain regions in patients with NDPH, including the left superior and middle frontal cortex, left precentral cortex, right lateral orbitofrontal cortex and insula. These areas involve processing and integrating pain and related emotions, affection, memory, and cognition.

It has been suggested that the frontal cortex is involved in the reorganization of the nociceptive network during pain processing of migraine [66, 67]. An observational study of brain morphology found a remarkable reduction in the frontal gray matter of migraine without and with aura compared with healthy controls [68]. Both functional and structural imaging studies indicated that the frontal lobe was the brain region strongly associated with migraine [66, 68]. Certainly, the brain structural study in adolescents with NDPH showed diminished cortical thickness in the left superior and middle frontal gyrus areas. There was a correlation between the thinned cortical thickness of the left superior frontal gyrus and headache sensitivity [11]. In another multimodal imaging study, NDPH showed markedly reduced cortical thickness in the rostral aspect of the left middle frontal gyrus and dwindled the cortical volume in the left superior and middle frontal gyrus regions. Magnetoencephalography revealed abnormal high-frequency cortical activity in the frontal cortex of NDPH [69]. Morphological investigations have identified reduced gray matter volume in the frontal cortex of patients with NDPH; however, it is unclear which specific cell types contribute to this observation. Our findings indicated a marked reduction in dendritic structures within the frontal cortex, which may account for the reduction in grey matter. It implies that NDPH is associated with neuronal hyperexcitation, potentially resulting in dendritic degradation or adaptive changes.

Evidence from many clinical studies supports the critical role of the primary motor cortex in the pain perception modulation. The voxel-based morphometric research demonstrated that migraineurs presented a significant focal gray matter loss in the left precentral gyrus [70]. However, no studies have been reported on the precentral gyrus in NDPH. We reported that the dendrite was reduced in the precentral cortex, which might be associated with pain processing and modulation. Motor cortical stimulation (MCS) has been applied to manage neuropathic and central pain. It has surfaced as a promising technique. The high-frequency repetitive transcranial magnetic stimulation of the left primary motor cortex modulates pain-related elicited responses in the patients with migraine [71]. Another randomized controlled trial of the transcranial direct current stimulation (tDCS) in the primary motor area in migraineurs has been proven effective in the prevention and treatment of both episodic and chronic migraine [72, 73]. These pieces of evidence have suggested that the precentral cortex is involved in the pathogenesis of headaches. What’s more, the ability to effectively relieve and treat pain through the non-invasive physical stimulation of the precentral cortex undoubtedly offered an optional treatment for patients with NDPH. We found dendritic loss in the left precentral cortex in the NDPH group, providing direct in vivo evidence of cortical microstructural abnormity for neuromodulation therapy. The precentral cortex may be an important brain region for neuromodulation in NDPH. In the future, it will be necessary to design a prospective cohort study to validate the treatment effects before and after the precentral cortex by neuromodulation.

The insula of patients with NDPH showed a reduction of dendrites. The insular cortex is a unique but hidden lobe of the human brain. It has five to seven oblique gyri. The central insular sulcus divides it into the anterior and posterior insula. The anterior insula is mainly associated with the limbic system, which plays a role in emotion regulation, whereas the posterior insula is crucial for sensorimotor integration. The anterior and posterior parts of the insula also maintain functional connectivity [74]. The cortical structure of the insula is thought to be pain perception, emotion, descending pain regulation, cognition, and autonomic function. Thus, the insula is often called the “center of activity” of migraine [75]. Utilizing a novel network mapping technique, Matthew discovered that different neuroimaging features of migraine converged in a single brain network, including the insula, visual cortex, and hypothalamus [76]. Because the insula can process and integrate multiple types of information from frontal, temporal, parietal, and occipital regions, it acts more like a “cortical hub”, translating signals from alterations in the internal environment of migraine and other chronic pain into complex behavior. Surface-based analysis found diminished dendritic density in the right insula of patients with NDPH. The insula cortex is mainly responsible for integrating multiple sensory and perceptual information, which may also serve as a central region in NDPH.

### Study limitations and future directions

It is the original to use NODDI to examine the brain microstructure in patients with NDPH. The interpretation of our findings was limited due to need for histopathological confirmation. More data must confirm our preliminary findings, as the sample size was relatively small. There were also some adolescents in the NDPH group. Still, due to the practical limitations of the study, our control group did not include any adolescents, which increased the heterogeneity of the participants. The small sample size and patient heterogeneity may limit the generalizability of these results. Although this study identified abnormalities of neurites in the WM and cortical GM, neural circuits still need to be made clear due to limited imaging equipment and algorithms. It isn’t easy to draw causal conclusions from this study because of its cross-sectional design. Thus, the causal relationship between neurite loss and NDPH whether it is a precipitating factor or a resultant outcome remains an open question. Designing longitudinal research endeavors that delve deeper into this enigma is imperative. Such studies will be instrumental in elucidating the dynamic interplay between neurite health and the evolution of NDPH, ultimately contributing to a more comprehensive understanding of this complex condition.

## Conclusion

Our study showed a reduction of white matter and cortical neurites in patients with NDPH compared to HCs. In addition, the regions with microstructural changes in fiber tracts and cortex are mainly involved in pain, emotion, and cognition modulation. These brain regions with abnormal neurites may be potential targets for neuromodulation treatment in patients with NDPH.

## Data Availability

Upon request, data can be provided.

## Abbreviations

NDPH: New Daily Persistent Headache
HCs: Health Controls
DTI: Diffusion Tensor Imaging
NODDI: Neurite Orientation Dispersion And Density Imaging
TBSS: Tract-Based Spatial Statistics
SBA: Surface-Based Analysis
NDI: Neurite Density Index
ODI: Orientation Dispersion Index
ISOVF: Isotropic Volume Fraction
SLF: Superior Longitudinal Fasciculus
ILF: Inferior Longitudinal Fasciculus
CST: Corticospinal Tract
IFOF: Inferior Fronto-Occipital Fasciculus
WM: White Matter
GM: Gray Matter
VAS: Visual Analog Scale
HIT-6: Headache Impact Test-6
PHQ-9: Patient Health Questionnaire-9
GAD-7: Generalized Anxiety Disorder-7
PSQI: Pittsburgh Sleep Quality Index
MoCA: Montreal Cognitive Assessment
BBR: Boundary-Based Registration
TFCE: Threshold-Free Cluster Enhancement
FWE: Family-Wise Error
CSD: Cortical Spreading Depression
MCS: Motor Cortical Stimulation
tDCS: Transcranial Direct Current Stimulation
